# Practical Medicine

**Published:** 1880-03

**Authors:** 


### Practical Medicine.

Treatment of Typhoid and of Typhus by the Douche.—(*Le Progrès Médical*, Sept. 20, 1879.)

M. Marcowitz of Bucharest communicates to the International Medical Congress at Amsterdam the results of his observations in regard to the treatment of typhoid and typhus by the douche.

The conclusions at which he arrives are: 1. The douche allows of the regular method of refrigerant treatment of 12.15 severe typhoid cases (allowing two to seven or eight douches for each patient) according to the tendency that the temperature has to return to the same point as before the application, without occupying more than two or three hours a day. 2. The patient is more thorougly roused by the douche than by the cold bath, and is due as much to the shock, which acts reflexly upon the nervous centers regulating the heat supply and preventing their paralysis for some hours at least, as to the actual withdrawal of heat. 3. In regard to contra-indications, M. Marcowitz is able to state with Liebermeister that scarcely anything but intestinal hæmorrhage, and a great paralysis of the heart, accompanied by a small and feeble pulse is absolutely opposed to the administration of the douche. 4. Pulmonary congestion, so long as it does not depend directly upon cardiac paralysis, is to be regarded rather as an indication than a counter-indication for the use of the douche.

TREATMENT OF SUB-ACUTE AND CHRONIC DYSENTERY BY IPECACUANHA AND BISMUTH INJECTIONS.—(*The Lancet*, Oct. 4, 1879.)

Dr. Houghton has resided in Sarawak, Borneo, for the last sixteen years, and has held during that time the appointment of principal senior medical officer to the government hospital, where malarial fevers, dysentery, and all endemic tropical diseases, are admitted into the medical wards. He has found, after a very large experience, that besides general treatment local applications in subacute and chronic cases of dysentery, by bismuth injections into rectum, have succeeded wonderfully, and that a great many hopeless chronic cases have been in this way relieved. The subnitrate of bismuth, to the extent of half a drachm, is to be rubbed down with the same weight of powdered gum in two ounces of cold water, and the resulting solution is to be injected from one to three times a day, according to the severity of the case. It is essential that the enema should be retained. The severe tenesmus and tormina will thus be relieved in a very short time. Ipecacuanha will of course be given, preferably in doses of one scruple to one drachm, at intervals of eight to twelve hours, according to the case, when the rectum symptoms are urgent. Bismuth is a most effectual remedy, and the clinical records of some hundreds of cases speak most highly of its effect.

THE EARTH TREATMENT OF ABDOMINAL TUMORS.—(*The British Med. Journ.*, Sept. 27, 1879.)

Dr. Addinell Hewson, of Philadelphia, has successfully treated by earth-dressing several cases of abdominal tumors, where either ovariotomy has been indicated, or when the case has been considered hopeless. An account of his proceedings may be found in No. 1 of the *Medical Bulletin*, published in Philadelphia. The material he used was pure brick-clay of a yellow tint (due to the iron in it) and free from sand and grit. This was dried by a low fire or in the sun, care being taken not to let it be roasted, because in such a case its therapeutical action was destroyed. It was then rolled and sifted, and about a pound and a quarter mixed with sufficient water to constitute a thick paste was spread all over the affected part. To make it dry quickly he laid a towel over it to absorb the water: the towel was then removed, and in its place a layer of cotton-wool was smoothly spread on the paste for the purpose of hastening the drying, and of holding the clay when dry, in its place. The dressing was then completed by a simple bandage, and allowed to remain till it would fall off in from two to five days. He attributes the action of the earth to some chemical property, which also has a calming influence on the pain. One of his first patients was a gentleman who was suffering from abscesses and diffuse inflammation of the cellular tissue of the knee, and had made up his mind to have his leg amputated. He recovered the use of his limb and was completely cured. The other patients who derived benefit from his treatment were all women suffering from tumors of the uterus and broad ligaments; one of them had narrowly escaped ovariotomy. Another had for years suffered severe pain during the catamenia, caused by a fibro-cystic growth which involved the right ovary. In a third case, the patient had previously undergone ovariotomy, and induced electricity had been applied through the uterus. The tumor grew again after the operation, and proved to be in reality a fibrous tumor of the uterus; there were besides several points of hernial protrusion of the bowels, on the spot of the wound, and she had the appearance and size of a woman at full term. The earth-dressing was applied under very unfavorable circumstances, but still proved successful, reducing her in size and preventing the hernial protrusions of the bowels by contracting the cicatrix.

MEDICAL TREATMENT OF HÆMORRHOIDS.—(*The Virginia Medical Monthly*, August. 1979.)

Dr. E. T. Sabal submits to the Medical Brief for August the following:

℞ Iodoform (ʒi), 4 grams. Powder very fine in a mortar, and add

Pulv. opii (grs. xv), 1 gram.

Vaselin (ʒi), 4 grams.

Mix. Apply locally morning and night and after every action of the bowels, after first washing with warm and cold water. One drachm of tannin may also be added to the above to deodorize the iodoform. Keep the bowels open by the following formula:

℞ Magnesiæ sulph.

Magnesiæ carb.

Sulphur. præcip.

Sacchar. lact. āā. (℥ss), 16 grams.

Pulv. anis. (ʒij), 8 grams.

Mix. Dose, one or two teaspoonfuls mixed in water at bed-time.

TREATMENT OF DYSENTERY BY RECTAL INJECTION.—(*The Lancet*, Oct. 18, 1879.)

Dr. King, commenting upon this method of treatment, which has been lately recommended by Dr. Houghton, remarks that in a large proportion of cases the dysenteric mischief is situated at the lower part of the colon and rectum, and local treatment is therefore of importance. In such cases benefit ensues as a result of directly curative measures; but even when the chief site is higher up, the effort to soothe the mucous membrane of the rectum, and allay spasm of the sphincter ani, amply repays the attempt by affording rest not only to the diseased bowel, but to the patient generally. The attainment of this object is frequently much assisted by the action of bismuth used per rectum. The remedy may be given as an injection, merely suspended in mucilage in the case of children, and in adults where opium is being administered by the mouth, or forty grains may be combined with half a drachm of tincture of opium, and if intolerance of ipecacuanha by the mouth is encountered, two scruples of this drug may be added. In either case the bowel is to be washed out in as gentle a manner as possible with lukewarm water, the object being to simulate the soothing process of fomentation ; to procure, for contact with the drugs, a clean surface ; and finally to diminish the chance of loss of injection by overcoming beforehand the spasm produced on the first introduction of the tube. The cleansing, if carefully done, is not injurious, but proves of the greatest utility by removing mucus and irritating secretions, which, by their presence, excite tenesmus, a result analogous to the cessation of pain which may be witnessed after removal of a plug of tenacious mucus from the os uteri in chronic metritis. Bismuth and opium injections have also been found useful after the employment of injections of nitrate of silver.

ON THE TREATMENT OF DIPHTHERIA.—(*The Glasgow Medical Journal*, October, 1879.)

Dr. Diamond concludes that the exhausting nature of diphtheria is generally granted, and that stimulants and support are required from the very commencement, whilst depressants and depletion are entirely contra-indicated. A large proportion of the profession advocate the non-interference theory, whilst others again adopt an entirely opposite view in regard to treatment; the great majority adopt a middle course, however, and seek to destroy but not to remove the false membrane. For this purpose all sorts of caustics and disinfectants, from the ancient nitrate of silver to the modern salicylic acid, have been recommended. These remedies, however, have, according to Dr. Diamond, for the greater part, one objectionable feature in common. They are nearly all poisonous, and cannot be swallowed with safety ; or if innocuous, they are apt to be powerless. On the whole he prefers the tincture of perchloride of iron, either of full strength or slightly diluted with glycerine. He is also strongly of opinion that the false membrane should be destroyed, and if possible removed. In regard to tracheotomy, no opinion is offered, but Dr. Diamond suggests that when it has to be performed, advantage should be taken of the opening to clear away the membranes from the larynx and trachea, and to disinfect their seat.

THE TREATMENT OF PARALYSIS AGITANS.—(*Le Concours Médical*, Vol. i., No. 1, July, 1879.)

M. Hammond applies a continuous current to the spinal cord, great sympathetic, and muscles affected by this disease; at the same time he administers strychnia and phosphorus according to the following formula: Phosphate of zinc 0.15; extract of nux vomica 0.30; conserve of roses q.s. To make thirty pills. A pill to be taken twice a day. This treatment is furthered by a tonic regimen, and the avoidance of all mental or physical excitement. Hammond has had six cases of this nature, of which four were cured within two months. Trousseau had already recommended the use of strychnia alone in this affection. Prof. Charcot had tried various medicines, and amongst others, nitrate of silver, all of which he found to be useless, and he confines himself to the recommendation of continuous currents. The following rules should, according to Dr. Onimus, be attended to, whilst employing continuous currents: The upper part of the spinal cord should be first stimulated with a sufficiently strong current; the negative electrode being placed at the base of the skull, the positive one upon the cervical vertebræ, and superior cervical ganglion. If the paralysis is limited to the upper limbs, the positive electrode should be applied to the brachial plexus for a part of the sitting, the negative pole being left in its original position.

A PECULIAR AFFECTION OF THE GAIT IN HABITUAL DRUNKARDS.—(*Charité Annalen*, iv. Jahrgang; *Med. chir. Rundschau*, June, 1879.)

Dr. Westphal has observed a peculiar form of the disturbance of gait in cases of chronic alcoholism. The affection bears certain resemblances to that occurring in tabes, from which, however, it may be readily differentiated. The peculiarity consists in the fact that at every step the limb is raised very much from the hip-joint, whilst the lower part of the leg remains bent, and then falls as if in the act of stamping. This affection cannot be mistaken for that arising from paralysis of the peroneal muscles, as the characteristic dependence and dragging of the foot is entirely absent. In tabes, on the other hand, the extension is made at the knee. In the disturbance of gait here alluded to there is entire absence of any ataxy of the lower extremities in the dorsal region: the sensibility remains intact in every form, and the tendon reflex is present to the ordinary extent. It is remarkable that after taking alcohol the disturbance of gait is lessened for a short time. Dr. Westphal explains the affection as due to painful sensations of cramp in the calf and in the knee-joint.

